# Exploratory regression modeling for flood susceptibility mapping in the GIS environment

**DOI:** 10.1038/s41598-023-27447-0

**Published:** 2023-01-05

**Authors:** Wang Fenglin, Imran Ahmad, Martina Zelenakova, Assefa Fenta, Mithas Ahmad Dar, Afera Halefom Teka, Amanuel Zewdu Belew, Minwagaw Damtie, Marshet Berhan, Sebahadin Nasir Shafi

**Affiliations:** 1 School of Environmental Studies, China University of Geosciences, (Wu Han), Hubei Province China; 2 China University of Geosciences Press Co., Ltd, Wuhan, Hubei Province China; 3 Department of Hydraulic and Water Resources Engineering, Debre Tabor University, Debra Tabor, Ethiopia; 4 Department of Environmental Engineering, Faculty of Civil Engineering, Technical University of Kosice, 042 00 Kosice, Slovakia; 5 Integrated Watershed Management Programme, Department of Rural Development and Panchayati Raj, Government of Jammu and Kashmir, Srinagar, India; 6 Department of Computer Science, Woldia University, Weldiya, Ethiopia

**Keywords:** Environmental impact, Sustainability

## Abstract

Understanding the temporal and spatial patterns of flood in the Awash River basin, which is located in Ethiopia’s Afar region, is crucial. The Awash basin was picked because it is continuously in danger both spatially and temporally. The likelihood of flooding was assessed using eight independent variables: elevation, slope, rainfall, drainage density, land use, soil type, wetness index, and lineament density. Each constituent was assigned a weight based on its susceptibility to the danger, which was classified into four classifications. Exploratory regression analysis showed that the existing land use is the main factor influencing flood susceptibility. For the GIS domain, a total of 31 models were built using exploratory regression. Model number 31 was found to be the best fit model, having the highest Adjusted R^2^ value of 0.8 and the lowest Akaike’s Information criterion value of 1536.8. The spatial autocorrelation tool’s Z score and p-value for the standard residuals are, respectively, 0.7 and 0.4, indicating that they were neither clustered nor scattered. The geographic breadth of flood susceptibility and risk is thoroughly examined in this paper, as is the significance of spatial planning in the Awash basin.

## Introduction

Geospatial planning is necessary to lower the danger of floods, a natural hazard^1,2^. The best method for reducing flood disaster risks and promoting decision-making is geospatial technology^3–5^. To lessen flood damage, researchers have examined floods, created flood hazard and flood hazard risk maps, and analyzed floods^6,7^. Numerous scientists have recognized that hazard zoning has a role in spatial planning processes^8,9^. Fluvial flooding results from an increase in a stream or river’s water level that overflows into the surrounding area and coastal areas, while pluvial flooding results from excessive runoff from rainfall that causes a rapid rise in water levels^10^. It’s possible that excessive rain or snowmelt is to blame for the rise in water levels. Snowmelt floods can cause numerous flood events that cause losses in both lives and property. Studying the hydrology of cold regions and how snowmelt causes floods is essential to reducing the risk of flood disasters^11^.

Flood risk analysis incorporates flood hazard mapping, which makes it possible to accurately estimate the spatial extent of flood characteristics like velocity, depth, and frequency^12^. Flood hazard maps are important for flood management strategies because they accurately depict the geographic extent and distribution of flood hazards^13^. There has been a lot of work done in recent years to analyze, predict, and quantify floods and their effects on the world.

The methods of physically-based, empirical, and physical modeling are the three main approaches for producing a flood hazard map^14^. Even though physically-based models have largely replaced the physical modeling approach, some researchers still use real-world experiments to simulate flood event scenarios from the past and the future^14^. Experimentation is necessary for the physical modeling approach to validate the model’s performance in making predictions. As an alternative, numerical models continue to be important as long as they simulate or accurately depict the physical/real processes underlying a flow or flood occurrence^15^.

A number of empirical and physically based models that can forecast floods may need data from remote sensing. Through physical experimentation, the physical models are also able to evaluate the extent of past and future flood hazards. The empirical models can be used in conjunction with a variety of statistical and data-driven techniques. The statistical and data-driven approaches depend on hydrological, topographic, Digital Elevation Model (DEM), and geomorphology data that are occasionally acquired using remote sensing and processed in GIS^16^. The multi-criteria decision-making method (MCDM)^17^, the statistical methods^18,19^, the machine learning approach^20,21^, and the artificial intelligence (AI) are the three categories into which the empirical methods fall^22^.

In 16 West African countries in September 2009, 600,000 people were impacted by torrential rainfall and flooding. Burkina Faso, Senegal, Ghana, and Niger were the nations that were worst impacted. This incident occurred shortly after the 2007 floods in Mozambique and Ethiopia that caused more than a million people to lose their homes and over 500 fatalities, as well as the 2008 floods in Burkina Faso, Togo, Mali, and Niger^23^. The most recent instances of the rising flood danger in Africa include these incidents and the steadily rising number of individuals impacted by floods during the 2009–2010 rainy season, which totaled roughly 25,000 until April 20. In reality, both the number of individuals impacted by floods and the economic damage they inflict has significantly grown in recent decades^24^. The number of flood-related deaths that occurred in Africa between 1950 and 2009 is around 16,000^25^.

Developing countries like Ethiopia, however, do not frequently adopt geospatial planning^26^. Nearly 80% of the yearly precipitation in Ethiopia falls during the three months from June to September. Especially in topographically low areas, the majority of the country frequently suffers torrential rain, which causes towns to flood^27–29^. The finest tools for long-term catastrophe management and mitigation are geospatial techniques^30–32^.

The article’s major focus is an analysis of flood inundation in Ethiopia’s Awash basin. Seasonal rainfall mostly causes flooding in the Awash River Basin, according to Tsay^33^. The Awash River's flooding enlarges its floodplains, floods topographical lows, and severely harms towns^34^. The intensity of threats to the Awash basin varies over time and space and is ongoing. Coenraads’^35^ testimony states that during the rainy season, the Awash River Basin briefly floods upstream but remains flooded for months at a time downstream, which impacts the grain output on flood plains. Floods may become stronger and more frequent as a result of climate change. Researchers have demonstrated that there are more flash floods presently in Ethiopia^36^.

The government instituted flood control management to lessen the likelihood of flooding in the Awash River basin. Although there are still problems, the government implemented flood control management to lessen the threat of flooding in the Awash River basin. However, there are still problems that might make the flooding in this area worse. The Awash River basin frequently floods in the months of August and September after heavy rains in the eastern highland and escarpment regions. Numerous tributaries that drain the highlands eastward and flood the low-lying alluvial plains that line the Awash River’s course cause its water level to rise quickly. Some locations, like the marshes close to Lake Yardi and the region between the towns of Debel and Gewane, experience frequent, nearly seasonal flooding. Flooding along the Awash River was primarily brought on by excessive rainfall in the eastern highlands and escarpment regions of North Shewa and Welo, as opposed to heavy rain in the upper watershed areas.

According to estimates, flooding could occur throughout the entire area set aside for irrigation construction in the Awash Valley. A region of about 200,000–250,000 ha is vulnerable to flooding during the Awash River's high flows^37^. Therefore, Ethiopia needs to map its flood hazards to help with decision-making and land planning, especially in the Awash basin.

This study’s main objectives are to evaluate the geographic distribution of flood susceptibility and risk as well as the effects of various factors on the flooding of the Awash basin.

## Study area

**Figure 1:**
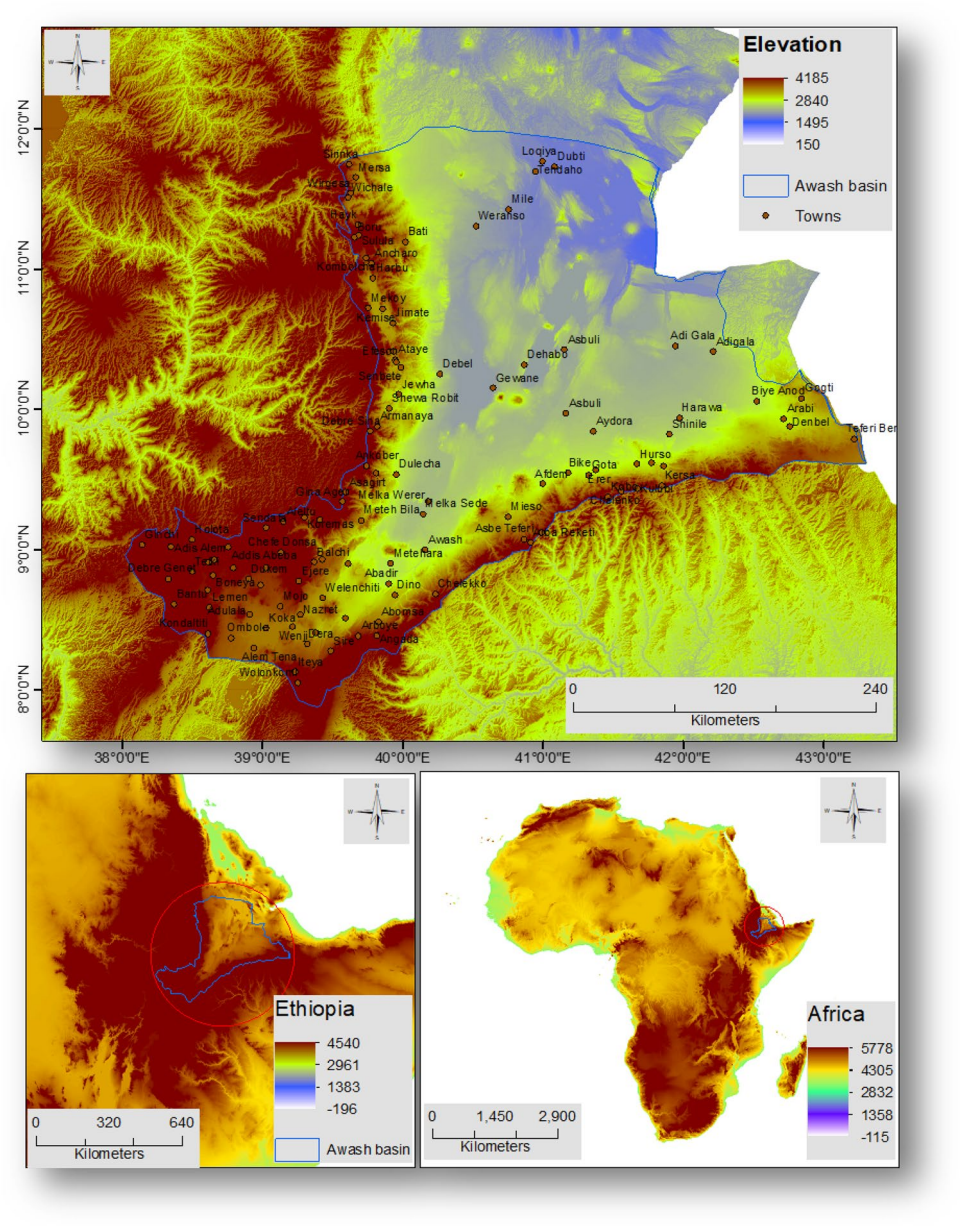
Location of the Awash basin, Ethiopia. This figure was created using ArcGIS 10.3.1 software.

The Awash basin in Ethiopia’s rift valley was chosen for the mapping of flood hazards. Between latitudes, 7°53'N and 12°N, and longitudes 37°57'E and 43°25'E, is where the Awash River Basin located^38^. The majority of the Awash River Basin, which is made up of dry lowlands, is located in northeastern Ethiopia's Afar Region. Between 362.5 and 2989.6 m are above the mean sea level in the Awash basin (Fig. 1). The study area is mountainous, undulating, and has slopes ranging from 0 to 46.38 degrees. The mean annual rainfall varies from approximately 178.6–461.1 mmyr-1 at Dubti, Tendaho, and Mile towns in the northeast to 1170.4–1452.8 mmyr-1 at Holota and Ginchi towns in the southwest of the study area. The lowlands of the Afar region make up the majority of the Awash basin. The Awash River rises in the basin’s southwest (close to Ginchi town), flows through the rift valley, and finally empties into Lake Abbe in the northern part of the basin. The river is 1202.0 km long in total. The river drains an area of land that is approximately 112,244.8 km^2^ and has a radius of 1994.4 km. The Indian monsoon and the Inter-Tropical Convergence Zone (ITCZ) both have a negligible but significant effect on the climate in the Awash basin^39^. The Awash Basin is divided into Upland (all lands above 1500 m above sea level), Upper Valley, Middle (area between 1500 and 1000 m above sea level), Lower Valley (area between 1000 and 500 m above sea level), and Eastern Catchment (closed sub-basin are between 2500 and 1000 m above sea level). The Upper, Middle and Lower Valley are all a part of the Great Rift Valleys systems. With a land area of 110,000 km^2^ and a population of 10.5 million, the Awash River Basin is the most significant in Ethiopia^40^. The river begins on a high plateau close to Ginchi Town on the western outskirts of Ethiopia’s capital city, Addis Abeba, flows through the Afar Triangle and the Rift Valley before coming to an end in the salty Lake Abbe at the border with Djibouti^41^.

The problem of river flooding brought on by a lot of rain falling quickly and the ensuing high river flow is a serious worry in the Awash River Basin, Ethiopia. The Awash River’s floodplain spreads to certain areas that are often dry throughout the main rainy season (June, July, August, and September). The low-lying, flat topography regions of the Awash River Basin are where the river or flash floods most regularly occur. Heavy rainfall falls on the mountains of the Awash River Basin, destroying communities along any section of the river and causing flooding downstream^34^.

## Methodology

Eight factors—elevation, slope, rainfall, drainage density, land use, soil type, topographic compound index, and lineament density—have been investigated and examined in the GIS field as potential determinants of flood susceptibility.

Downloaded from https://a sterweb.jpl.nasa.gov/gdem.asp were the Advanced Spaceborne Thermal Emission and Reflection Radiometer (ASTER)-DEM data relevant to the research region. ASTER-DEM has a spatial resolution of 30 m and 14 distinct bands, from ultraviolet to infrared. The research area’s slope and height were calculated using ASTER-DEM data. Additionally, a spatial analyzer tool was employed.

Data on precipitation (a combined satellite gauge-estimated amount) for the months of January through December 2021 were downloaded from Global Precipitation Measurements, which have a spatial and temporal resolution of 0.1^0^ and monthly, respectively. Empirical Bayesian Kriging (EBK) was used to project the annual precipitation data (mm) using the coordinate system for Adindan UTM Zone 37 N. Validity was examined for the EBK semivariogram prediction errors. Empirical Bayesian Kriging (EBK) was used because it automatically modifies parameters through sub-setting and simulations to provide reliable findings. Additionally, unlike other kriging techniques, EBK takes into consideration the mistake caused by calculating the underlying semivariogram. The EBK for precipitation is validated since the prediction errors for the mean standardized, root mean square standardized, and average standard for precipitation are, respectively, 0.01, 0.98 and 8.6.

To calculate the study area's river density map utilizing the natural breaks technique in order to find actual classes in the data. The Food and Agriculture Organization (FAO) (http://www.fao.org/soils-portal/soil-survey/soil-maps-and-databases/en/) provided a soil map (soil texture) of the research region. For the year 2021, a land use map of the study region was created using data from https://landsatlook.usgs.gov. The research region was divided into grids, and the length of the lineament in each grid was calculated to determine the lineament density. The lineament map was acquired from the Geological Survey of Ethiopia at a scale of 1:250,000.

The kriging interpolation technique was used to compute missing or no-value data in the factors. To accommodate the largest neighborhood, we choose the major semi-axis and minor semi-axis as 118,661.1 and 111,161.1, respectively, along with a tolerance of 45^0^. At least 18 nearby values with various weights were used to determine the unknown value. On a scale of 1 to 5, each factor’s weight has been determined (1, Very low hazard/susceptibility; 5; Very high susceptibility/hazard).

### Each factor was reclassified using the “Natural Breaks” classification in the GIS domain

Each topographical class has been assigned a weighting based on how susceptible it is to flooding. Low-lying areas, such as those between 362.5 and 960.04 m, have been given the highest weight of 5, because they are more susceptible to flooding, while areas between 2309.6 and 2989.6 m, at the highest elevation, have been given the lowest weight of 1 because they are the least susceptible to flooding.

Since flat to gentle slopes (0–4.06^0^) are more susceptible to floods, they have been given the highest weighting of 5, or 5. The flood potential estimate for steep slopes (20.28 to 46.38^0^) is 1, indicating that they are the least susceptible to floods.

Rainfall between 178.6 and 461.1 mm was given the lowest weight of 1, and rainfall between 1170.41 and 1452.86 mm was given the maximum weight of 5.

The greatest weight of 5 has been given to drainage density since it contributes the most to flooding, specifically between 5 and 9.56 km/km^2^. However, because low drainage densities are typically found at high elevations, drainage densities between 0.08 and 1.16 km/km^2^ have been given the lowest weight of 1.

In terms of land use, the classifications of the forest, woodland, and afro-alpine land use received the lowest potential scores of 1. While the maximum weight of 1 has been given to land use types like settlements, wetlands, and water bodies. A weight of 4 was provided to land use classes like urban and bare land, and a weight of 3 was given to land use classes like grassland and agricultural land.

Pellic and chromic vertisols have been given the maximum weightage of 5 for the research area’s soil type, suggesting that they have the most contribution to flooding due to their low infiltration rates (thus much water will be available on the land as an overland flow). Contrarily, due to their reduced susceptibility to flooding, chromic cambisols, and eutric nitisols have been given the lowest weight of 1, or 1. (due to their higher infiltration rates very less water will be available as an overland flow).

The areas with CTI values between 1.46 and 8 have been assigned the highest weighting of 5, in the case of CTI. However, because of their lowest potential for wetlands, locations with CTI values between − 0.02 and − 0.16 were given the lowest weight of 1.

Lineament density (Ld) has been determined using ArcGIS 10.3.1 and is the total length of the lineaments per unit area of the research region. Therefore, the lowest weight of 1 was given to locations with the highest Ld values (6.8 to 12.9 km/km^2^). Areas with the lowest Ld values (0.16 to 0.43), nevertheless, received the greatest weight of 5.

Each element was divided into five classes for flood danger and risk, and weights were assigned to each class in the GIS domain (Table 1). To create the flood danger map, all of the weighted components were permitted to overlay in the GIS domain. The flood risk raster map has been divided into 5 categories, including Very low (1), Low (2), Moderate (3), High (4), and Very High (5) hazards. The weighted population distribution map and the land use map were overlaid with the flood susceptibility map to calculate the flood risk map.

**Table 1:** Thematic layers, their areal extent along with their potential values and the associated hazard.

Theme	Classes	Area (%)	Potential value	Hazard
Elevation	2309.68–2989.64	7.25	1	Very low
	1938.79–2309.67	15.90	2	Low
	1506.09–1938.78	18.68	3	Moderate
	960.05–1506.08	23.76	4	High
	362.5–960.04	34.41	5	Very high
Slope	0–4.06	49.32	5	Very high
	4.07–6.36	31.05	4	High
	6.37–10.11	13.09	3	Moderate
	10.12–20.27	5.21	2	Low
	20.28–46.38	1.34	1	Very low
Rainfall	178.67–461.13	26.65	1	Very low
	461.14–707.94	22.89	2	Low
	707.95–923.59	17.93	3	Moderate
	923.6–1170.4	18.95	4	High
	1170.41–1452.86	13.58	5	Very high
Drainage density	0.08–1.16	2.60	1	Very low
	1.17–1.69	11.68	2	Low
	1.7–2.77	25.12	3	Moderate
	2.78–4.99	38.51	4	High
	5–9.56	22.08	5	Very high
Land-use	Forest, Woodland, Afro-alpine	3.67	1	Very low
	Grassland, Shrubland, Plantation	40.64	2	Low
	Cultivation land	21.70	3	Moderate
	Bare land, Urban land	32.70	4	High
	Water bodies, Settlements, Wetlands	1.29	5	Very high
Soils	Chromic cambisols, Eutric nitisols	21.43	1	Very low
	Calcaric regosols	36.76	2	Low
	Lithosols	5.37	3	Moderate
	Fluvisols, Yermisols	3.44	4	High
	Pellic and chromic vertisols	32.99	5	Very high
Topography index	− 0.02–− 0.16	10.54	1	Very low
	− 0.17–0.19	8.72	2	Low
	0.2–0.37	22.33	3	Moderate
	0.38–1.45	34.38	4	High
	1.46–8	24.03	5	Very high
Lineament density	6.81–12.91	3.35	1	Very low
	0.44–1.49	6.09	2	Low
	1.5–3.39	13.49	3	Moderate
	0.44–1.49	30.62	4	High
	0.16–0.43	46.44	5	Very high

To obtain the final spatial distribution of flood danger hotspots, all independent variables (factors) were overlaid in the GIS domain. In ArcMap 10.3.1, we utilized the Exploratory Regression tool to examine how each element affected the sensitivity to floods. According to the maximum variance inflation values of the models (7), there is no exploratory variable redundancy in any of them. It is crucial to confirm that the standard residuals are normally distributed after doing regression modeling. The model must be biased or invalid if the standard residuals are clustered or dispersed. Therefore, the standard residuals were subjected to the spatial autocorrelation tool (Moran’s I). To create a flood risk map for improved spatial planning, the flood susceptibility map has also been superimposed on a population map of the research area. The following are the important steps used in this study:

- Creation of determinant factors of flood susceptibility.
- Assigning suitable flood potential values/weights to the factors.
- Weighted overlay of factors to get the flood potential map.
- Creation of flood risk map by integrating population density factor.
- Exploratory regression modeling between the determinant factors with respect to the flood potential map.
- Conducting the Spatial autocorrelation (Global Moran`s I) to check if the residuals follow the Gaussian pattern.

**Figure 2:**
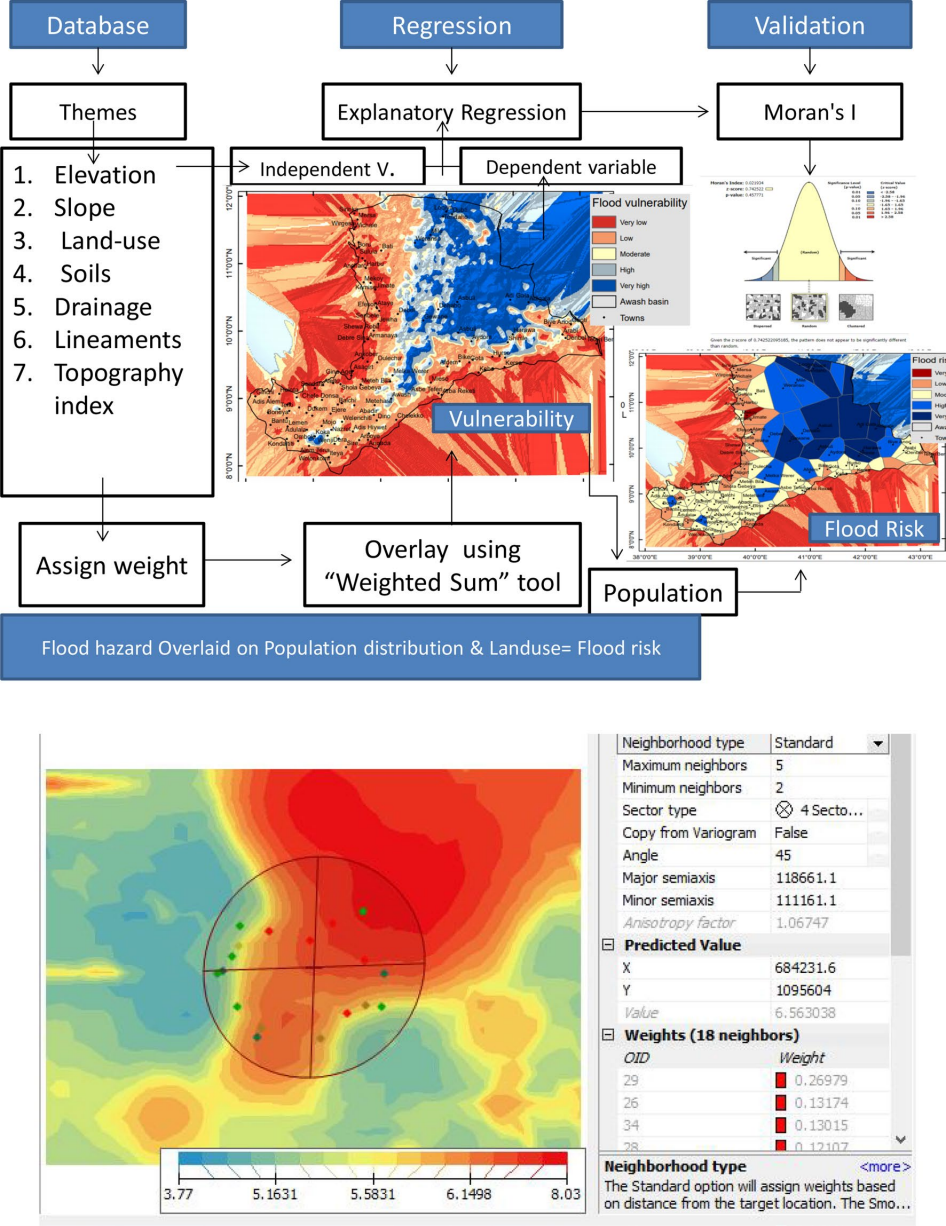
Methodology employed. This figure was created using ArcGIS 10.3.1 software.

Figure 2 displays the overall methods followed in this investigation.

## Results

### Determinant factors

#### Elevation and slope

**Figure 3:**
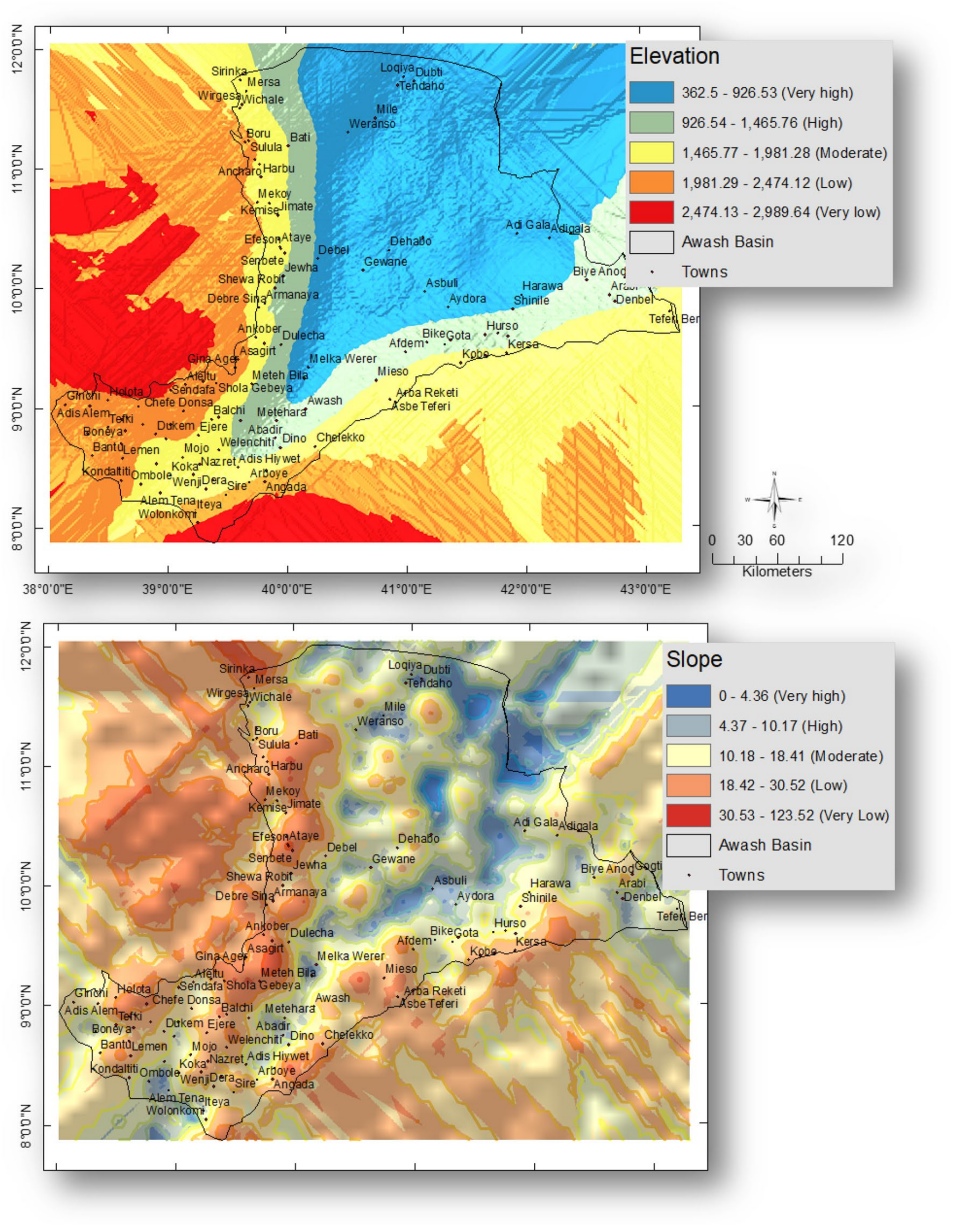
Weighted layers of elevation (meters) and Slope (degrees) of the Awash basin, Ethiopia. This figure was created using ArcGIS 10.3.1 software.

The elevation of the land determines the flood’s velocity and direction. The research area's elevation ranges from 362.5 to 2989.64 m. The Awash basin's total area was found to be 34.41% covered by topographic lows (362.5–960.04 m). Only 7.25% of the study region was determined to be covered by the topographic highs (2309.6–2989.6 m). Figure 3 displays the elevation's weighted map. Another aspect that affects whether there will be a flood is the slope. The study region has a slope that varied from 0 to 46.38^0^. In comparison to steep slopes, flat to gentle slopes are more susceptible to overland flow buildup. The majority of the research area (49.32%) has flat to mild slopes as its subsurface (0–4.06^0^). Figure 3 displays the slope’s weighted map.

#### Rainfall and drainage density

**Figure 4:**
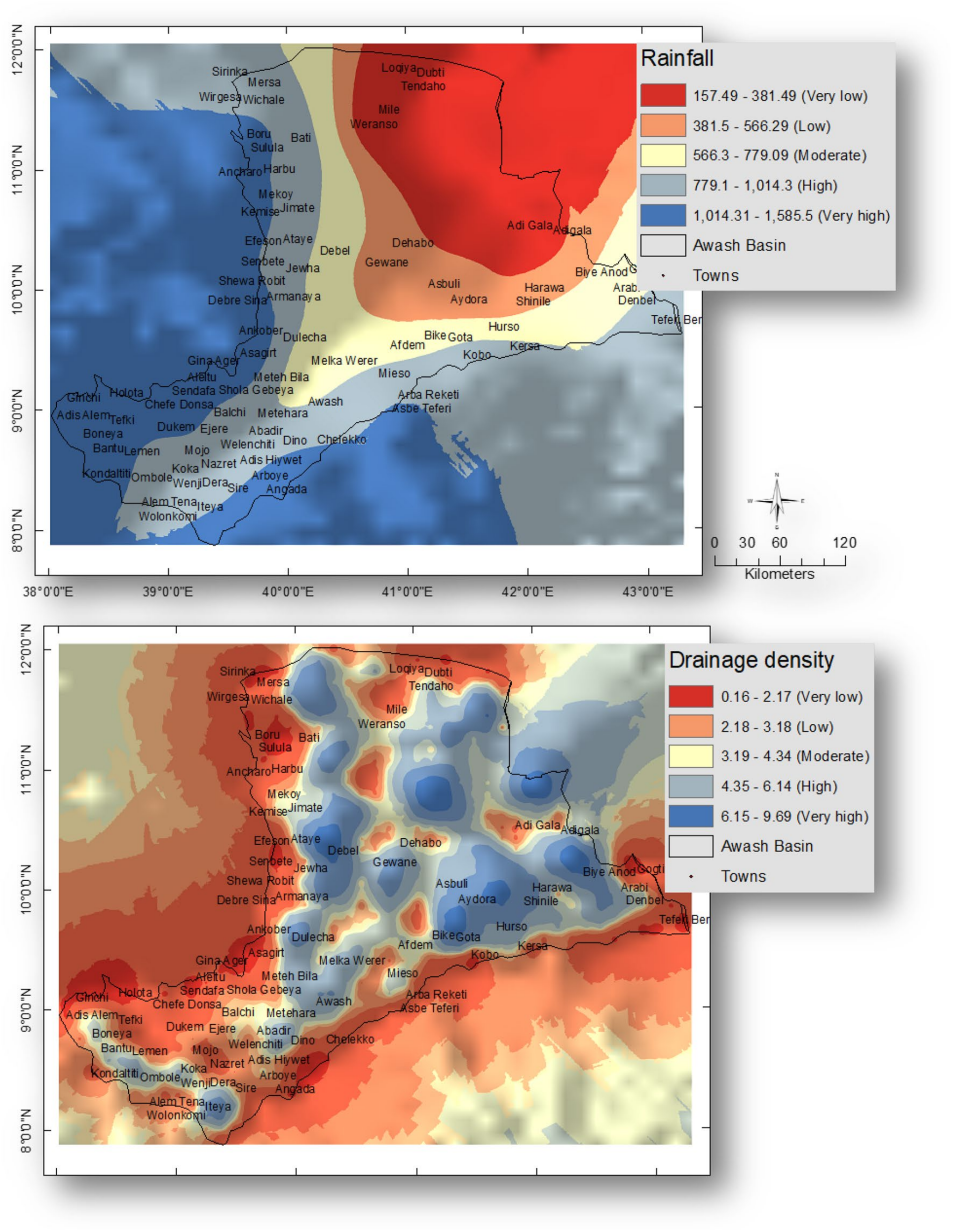
Weighted layers of Rainfall (mm) and Drainage density (km/km^2^) of the Awash basin, Ethiopia. This figure was created using ArcGIS 10.3.1 software.

Another significant aspect affecting flood susceptibility is rainfall (178.6–1452.8 mm). The research region was found to be covered by 13.5% of the highest rainfall (1170.4–1452.8 mm), and 26.6% of the lowest rainfall (178.6–461.1 mm). Any place that receives more rainfall is more susceptible to flooding^42^. Different rainfall types have been given an appropriate weighting by their risk for flooding. Figure 4 displays the weighted rainfall map. The region’s drainage density varies from 0.08 to 9.5 km/km^2^. The highest Dd values (5–9.5 km/km^2^) cover about 22.08% of the region. The research area’s drainage densities have been categorized by their potential for flooding hazards. Figure 4 displays the weighted drainage density map.

#### Land use and soils

**Figure 5:**
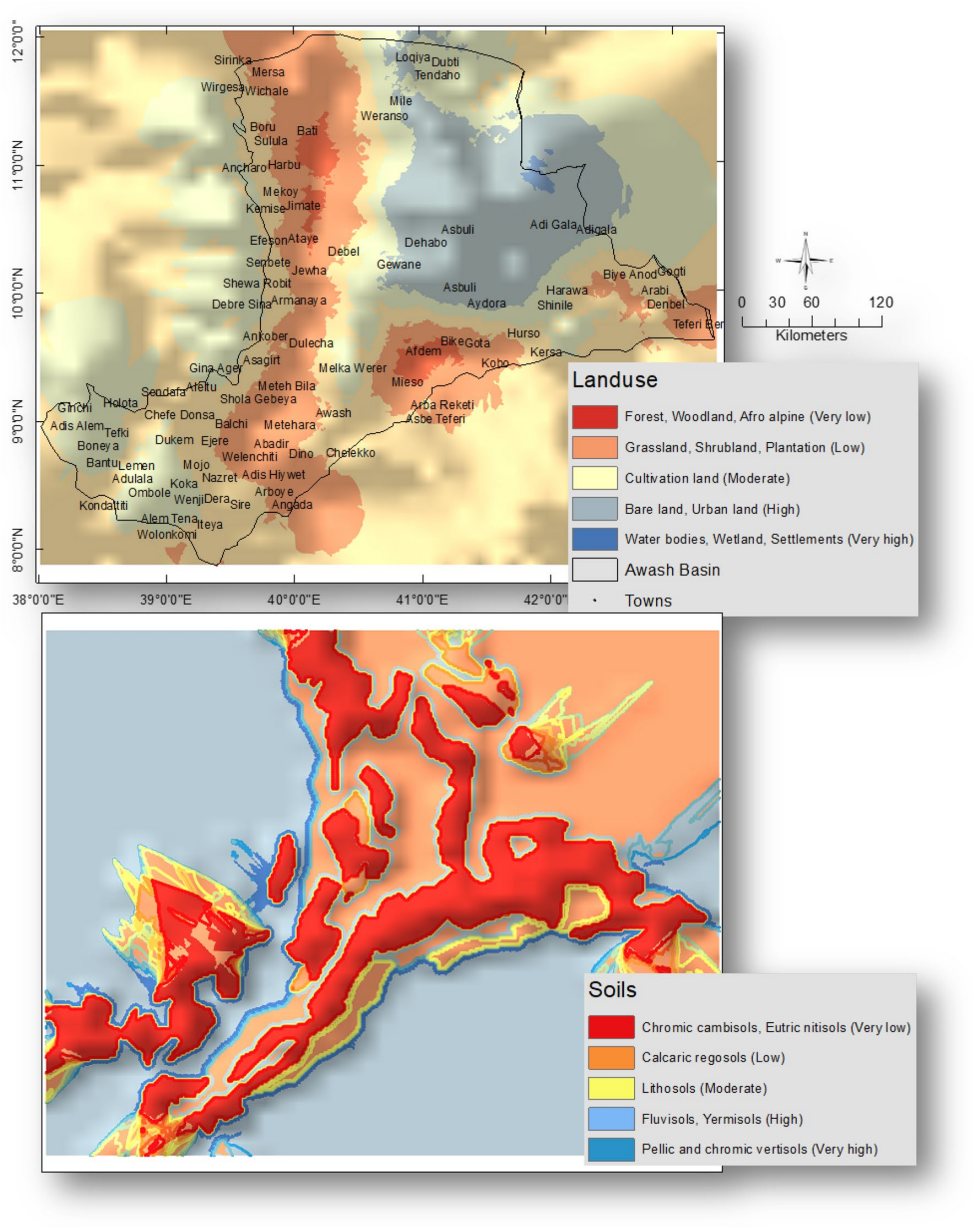
Weighted land use and soils of the Awash basin, Ethiopia. This figure was created using ArcGIS 10.3.1 software.

Wetlands, populated areas, and water bodies were determined to make up 1.2% of the entire research area. 3.6% of the total area is calculated to be covered by forest, woodland, and afro-alpine. The study area’s land use map was examined in light of its susceptibility to flooding. The various land use types have therefore been given the appropriate weighting. Figure 5 shows the weighted land use map. The permeability, porosity, and infiltration rates of various soils vary. The potential of the soil in terms of flooding is determined by its soil properties^43^. As a result, the various soil types in the research region have been assigned the appropriate amount of weight. Figure 5 displays the research area’s weighted soil map.

#### Topography index and Lineament density

**Figure 6:**
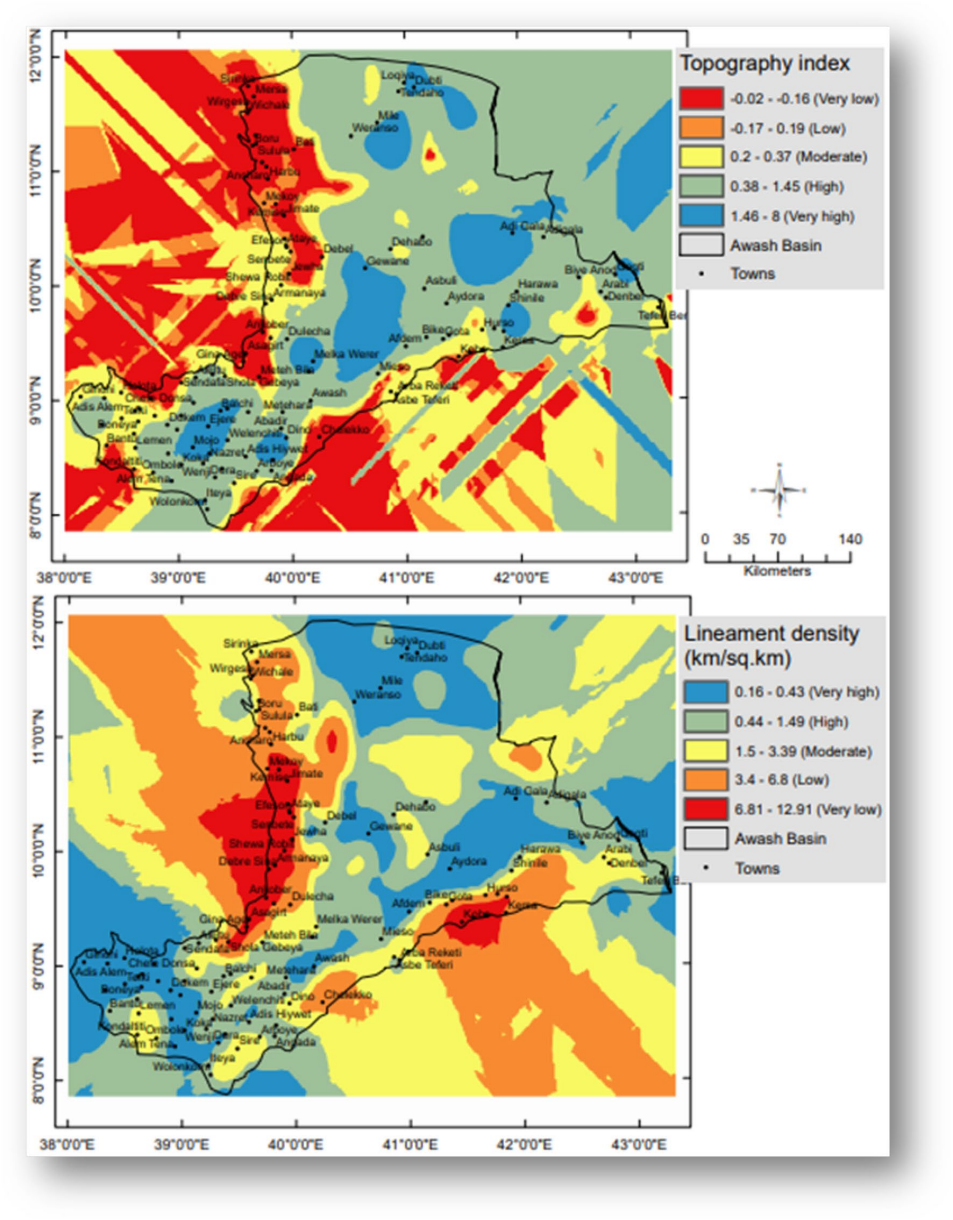
Weighted topographic wetness index and lineament density of the Awash basin, Ethiopia. This figure was created using ArcGIS 10.3.1 software.

In the GIS domain, the Compound Topography Index (CTI) of the research area has been calculated. The study area's CTI values range from − 0.02 to 8. According to calculations, the areas covered by the greatest CTI values (1.4–8) account for 24.03% of the entire area. Compared to places with lower CTI values, those with higher CTI values offer greater potential for wetlands. In Fig. 6, the weighted CTI map is displayed.

The research area’s lineament density, Ld, ranges from 6.8 to 0.4 km/km^2^. There are more pathways for water to infiltrate downhill in areas with greater Ld levels. Figure 6 displays the Ld’s weighted map.

#### Flood hazard and flood risk

Very low, low, moderate, high, and very high flood zones each cover an area of 6.96, 10.21, 33.09, 38.28 and 11.46 km^2^ turn.

**Figure 7:**
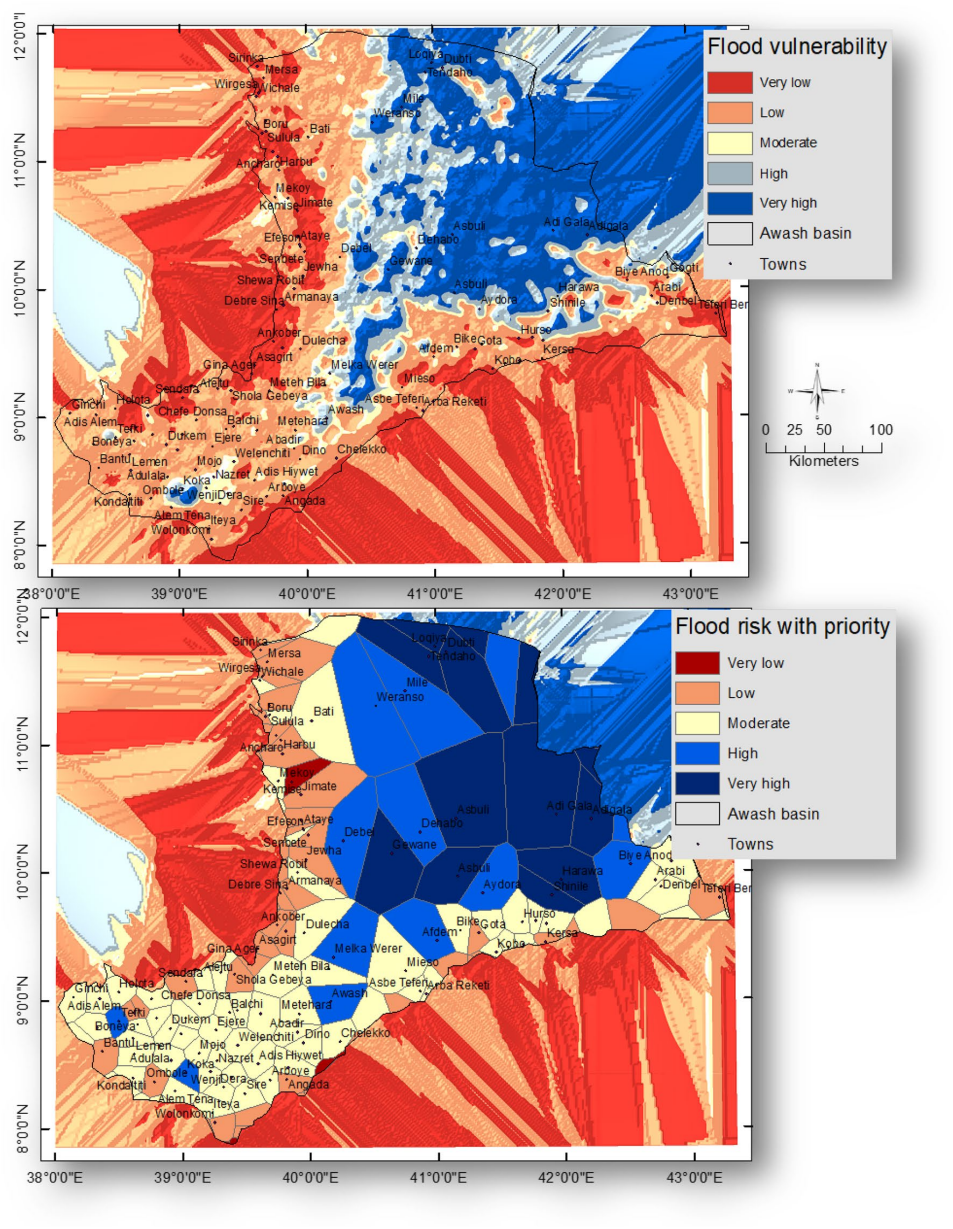
Spatial distribution of flood vulnerability and flood risk map of the Awash basin, Ethiopia. This figure was created using ArcGIS 10.3.1 software.

Accordingly, the output flood risk map was ranked to determine which regions should receive preference for flood management procedures. Figure 7 displays the flood susceptibility and flood risk map. Due to their high level of flood susceptibility, places including Adi Gala, Asbuli, Gewane, Loqiya, Weranso, and Mile have been given priority during the management process. However, because they have “extremely low” flood risks, towns including Alem Gena, Sendafa, Gina Ager, Ankober, Assagirt, Aliyu Amba, Debre Sina, Armanaya, Jimate, Ancharo, Karakore, Kombolcha, Boru, Wirgesa, Sirinka, Kobo, Hurso, Gota, and Teferi Ber had the lowest priority for flood control. Table 2 displays the area of each flood hazard zone, the related municipalities, and their priority.

**Table 2:** Areal extent of flood vulnerability in the Awash basin, Ethiopia.

Flood hazard	Area (Km^2^)	Percent	Representative towns	*Priority
Very low	7777.28	6.96	Alem Gena, Sendafa, Gina Ager, Ankober, Assagirt, Aliyu Amba, Debre Sina, Armanaya, Jimate, Ancharo, Karakore, Kombolcha, Boru, Wirgesa, Sirinka, Kobo, Hurso, Gota, and Teferi Ber	5
Low	11,414.23	10.21	Bantu, Melka Kunture, Dukem, Debre Zeyt, Enjere, Holota, Iteya, Sire, Angada, Balchi, Koremas, Chelekko, Meteh Bela, Arba Reketi, Kersa, Arabi, Gogli, Mersa, Hayk, and Kemise	4
Moderate	36,975.46	33.09	Harbu, Bati, Mekoy, Shewa Robit, Debel, Dulecha, Metehara, Lemen, Kondaliti, Arboye, Wolonkomi, Bike, Shinili, Dire Dawa, Dehabo, Wenji, Nazeret, Sodore, Alem Tena, and Ambosa	3
High	42,773.32	38.28	Harawa, Aydora, Dubti, Melka Werer, Awash, Koka, and Tendaho	2
Very high	12,801.18	11.46	Adi Gala, Asbuli, Gewane, Loqiya, Weranso, and Mile	1

*Priority: 1–5; where 1 means the highest priority and 5 means the lowest priority.

## Discussion

**Figure 8:**
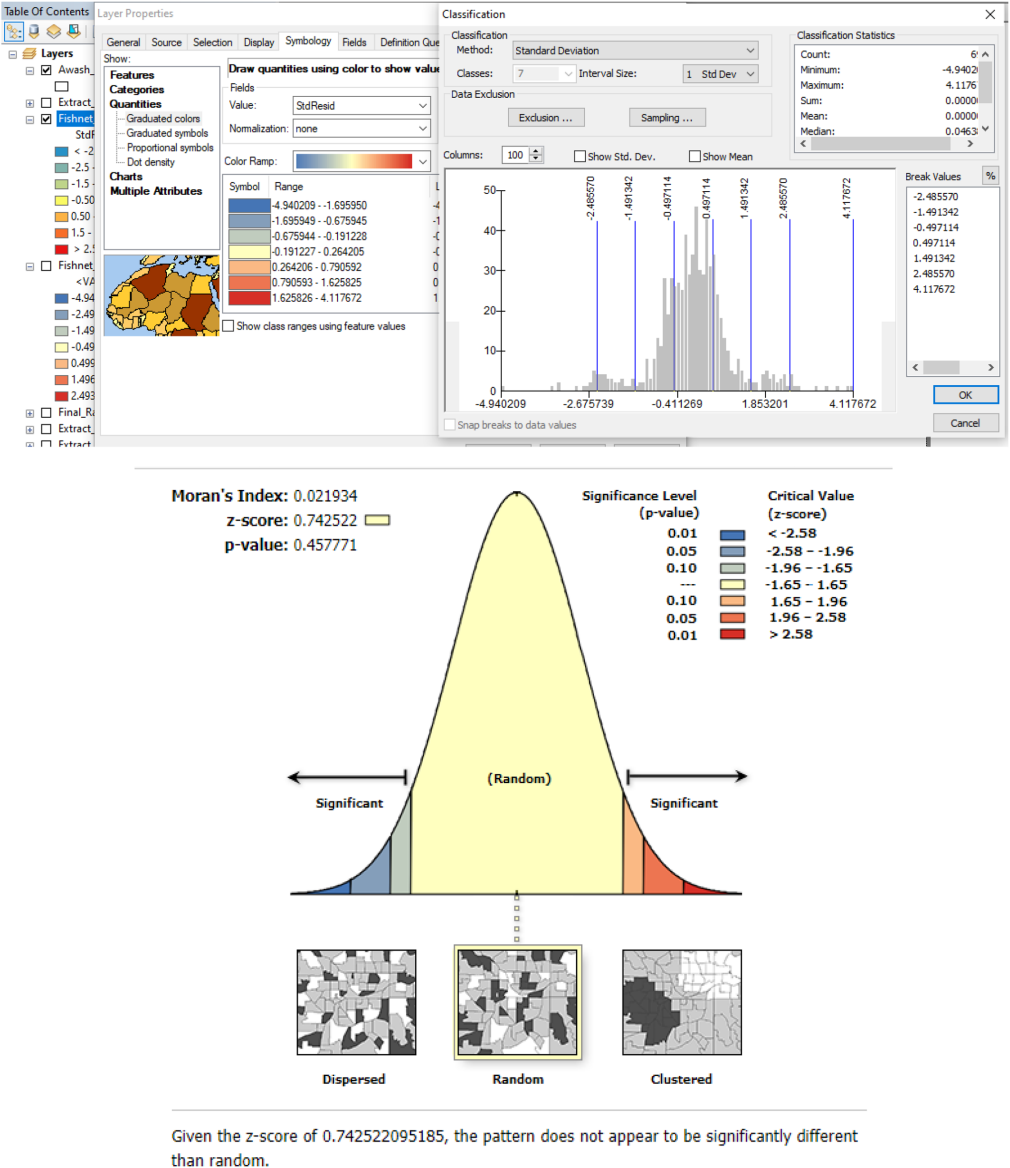
Distribution of standard residuals of exploratory regression. This figure was created using ArcGIS 10.3.1 software.

In the GIS domain, each thematic layer has been given a weight based on how susceptible it is to erosion. The flood susceptibility and risk map make it evident that the study area's areas most dangerous are located in its northeastern corner, which is located in topographic lows. Different determinants may have varying effects on flood susceptibility. Exploratory regression analysis was carried out in the GIS domain to comprehend the impact of several determinant elements on the hotspots of flood hazard potential. To rule out any variable having collinearity or redundancy, correlations between the variables must be performed before performing the exploratory regression. Land use had the highest adjusted R value among the eight components, with a value of 0.4, followed by rainfall (0.37), elevation (0.31), lineament density (0.1), topographic index (0.06), slope (0.05), and drainage density (0.03). The study area's current land use has the greatest influence on the likelihood of flooding, with drainage density having the least impact. As a result, a change in cultivation in the research region of just one unit may result in a 41.1% increase in risk. All seven variables had Koenker (BP) values that were not statistically significant (p > 0.005). The factor with the best match, as indicated by its lowest Akaike’s Information criterion value of 1872.9, is land use. To obtain the best model fit, 31 alternative combinations of the separate components were created. Additionally, none of the models’ Koenker’s (BP) statistics are statistically significant (p > 0.005). Model number 31 was discovered to have the best model fit out of all the other 30 models, as evidenced by its lowest Akaike’s Information criterion score of 1536.8. (Table 3). The drainage density element wasn’t included in model number 31 because it didn’t have a significant impact on the model. It is crucial to confirm that the standard residuals are normally distributed after doing regression modeling. The model must be biased or invalid if the standard residuals are clustered or dispersed. Therefore, the standard residuals were subjected to the spatial autocorrelation tool (Moran’s I). It was discovered that the standardized residuals were dispersed at random (Fig. 8). With a Z score and p-value of 0.7 and 0.4, respectively, Moran’s index value of 0.02 was obtained.

**Table 3:** Exploratory regression models.

Model	AdjR^2^	AICc	JB	K-BP	MaxVIF	X1	X2	X3	X4	X5	
1	0.3762	1913.932	0.152	0.412	1.000	Rf					
2	0.035779	2216.597	0.174	0.496	1.000	Dd					
3	0.41192	1872.95	0.132	0.008	1.000	Lc					
4	0.104506	2165.205	0.144	0.102	1.000	Ld					
5	0.062832	1873.78	0.167	0.232	1.000	Cti					
6	0.314543	1979.439	0.165	0.109	1.000	Elev					
7	0.05857	2199.972	0.199	0.090	1.000	Slp					
8	0.379233	1911.564	0.188	0.477	1.199	Rf	Dd				
9	0.621215	1568.243	0.183	0.008	1.078	Rf	Lc				
10	0.383913	1906.304	0.196	0.137	1.180	Rf	Ld				
11	0.439723	1840.309	0.197	0.070	1.001	Dd	Lc				
12	0.128952	2146.988	0.186	0.002	1.011	Dd	Ld				
13	0.318867	1976.061	0.193	0.090	1.266	Dd	Elev				
14	0.076497	2187.63	0.171	0.073	1.060	Dd	Slp				
15	0.47169	1799.479	0.171	0.089	1.016	Lc	Ld				
16	0.614277	1580.857	0.151	0.076	1.036	Lc	Elev				
17	0.424097	1859.427	0.192	0.080	1.045	Lc	Slp				
18	0.341823	1952.234	0.199	0.065	1.094	Ld	Elev				
19	0.238	1953.543	0.178	0.048	1.012	Ld	Cti				
20	0.126371	2149.045	0.149	0.078	1.105	Ld	Slp				
21	0.627226	1558.149	0.192	0.083	1.253	Rf	Lc	Ld			
22	0.628398	1555.961	0.192	0.471	2.659	Rf	Lc	Elev			
23	0.492162	1773.036	0.188	0.453	1.026	Dd	Lc	Ld			
24	0.444778	1835.035	0.192	0.347	1.106	Dd	Lc	Slp			
25	0.345323	1949.552	0.193	0.479	1.372	Dd	Ld	Elev			
26	0.14216	2137.393	0.181	0.387	1.160	Dd	Ld	Slp			
27	0.629715	1553.492	0.299	0.451	1.122	Lc	Ld	Elev			
28	0.5432	1544.362	0.289	0.442	1.134	Lc	Ld	Cti			
29	0.636866	1540.968	0.382	0.441	2.204	Rf	Lc	Elev			
30	0.732728	1548.844	0.398	0.498	1.222	Lc	Ld	Elev	Slp		
**31**	**0.839539**	**1536.866**	0.432	0.421	2.353	Rf	Lc	Ld	Elev	Slp	Cti

Significant values are in bold.

Where, *AdjR*^*2*^ Adjusted R squared, *AICc* Akaike’s information criterion, *JB* Jarque-Bera statistic, *K-BP* Koenker (BP) statistic, *MaxVIF* Maximum Variance Inflation, *X* variables.

*Rf* rainfall, *Dd* drainage density, *Lc* landcover, *Ld* lineament density, *Elev* elevation, *Slp* slope, *Cti* compound topography index.

Measures of model performance.

When this test is statistically significant (p < 0.01) model predictions are biased (the residuals are not normally distributed).

When this test is statistically significant (p < 0.01), the relationships modeled are not consistent (either due to non-stationarity or heteroskedasticity).

Large VIF (> 7.5, for example) indicates explanatory variable redundancy.

In the Awash River Basin, Ethiopia, the issue of river flooding caused by excessive rainfall in a short period and the subsequent high river flow is of major concern. The major rainy season (June, July, August, and September) is when the Awash River's floodplain stretches to specific locations that are generally dry. The Awash River Basin’s low-lying, flat topographic sections are where the river or flash floods most frequently occur. The mountains of the Awash River Basin receive heavy rainfall, which ruins towns along any stretch of the river and causes flooding downstream^34^. The yield of the crops grown on the floodplain will depend on the frequency and magnitude of the flood. In the Upper Awash River Basin, heavy rains at the beginning of the rainy season will create flooding and leave behind fertile sediment in the floodplain. Floods can harm crops if the Upper Awash River Basin receives a lot of precipitation near the end of the rainy season. In many ways, floods are becoming much more unpredictable^35^.

Due to crop damage and costs to human welfare, flooding is becoming a major concern in the Awash River Basin, making GIS-based flood hazard assessment and extent mapping essential. In the Awash River Basin, there is a need for flood control, accurate forecasts, and hazard extent mapping. According to our research, the inundated areas in the Upper and Middle Awash River Basin are typically lower than those further downstream.

Knowing which locations are at risk of floods will improve emergency response efforts. To prepare local communities for an emergency, it is possible to identify locations that would likely need to be evacuated and develop and properly indicate evacuation routes. Finding flood shelters for evacuees will be made easier with the identification of flood risk regions. A more effective emergency response may likely be planned with the identification of flood risk locations. During a flood occurrence, it is crucial that key infrastructure, such electrical supply, sewage treatment, etc., and services, like the emergency services, continue to run. So that they may continue to function during a severe storm, planners will be able to place these components in low-risk zones using flood hazard maps. As an alternative, flood hazard mapping may emphasize the need to protect these components from flooding.

Before the full advantages can be realized, flood hazard mapping must be integrated into other processes, such emergency response planning and town planning, in order to reduce flood risk. Due to a shortage of data from witnessed severe events, more sophisticated, reliable flood danger maps are likely to rely on complicated numerical models. To accomplish this, a certain level of knowledge is needed. It could be expensive to gather topographic and bathymetric data to supplement information on exceptional water levels and wave heights.

One limitation of this strategy for studying flood hazards and risks using GIS is the absence of an appropriate hydraulic approach for calculating stages in the GIS output. Therefore, no flood depth inundation estimates or hydrodynamic models were employed in the inquiry. Future research using hydrodynamic modeling and GIS might look at flood depth inundation.

## Conclusion

Eight determining factors in all were picked to examine the flood risk hotspots in Ethiopia’s Awash basin. Each variable was weighted based on how likely it was to flood. To create the spatial maps of flood risk and susceptibility, all the weighted components were layered. Very low, low, moderate, high, and very high hazard zones have respective area extents of 6.96, 10.21, 33.09, 38.28 and 11.46 km^2^. The output map shows that the northeastern portion of the research area is in a zone with “extremely high” flood potential and danger; as a result, it requires priority in terms of spatial planning. The low-lying areas close to the Awash River, especially in the downstream portion, are in the high to very high flood susceptibility zone, according to the flood hazard assessment. The Awash River Basin has flood hazard concerns in the high to very high range in the north, northeast, parts of the southwest, and west escarpments. especially agricultural land in low-lying locations with a high or very high danger of flooding. The heavy rains during the primary rainy season are the greatest contributor to flooding in the low-lying area along the Awash River. As a preliminary information source for land use planning and spatial policymaking, the study’s findings are crucial. To show which areas deserve the highest and lowest priority, a priority table has been created. Exploratory regression demonstrated that the primary factor affecting the flood potential is the current land usage. The model was validated by the spatial autocorrelation tool, which revealed the standard residuals’ random distribution. This study demonstrated the effectiveness of the exploratory regression model as the best way to comprehend the impact of multiple determining thematic layers on the mapping of flood susceptibility and flood risk. With the minimal adjustment, this technique may be utilized at the continental level.

### Ethics approval and consent to participate

We all authors approve the paper.

## Data Availability

The data will be provided based on a request (to the corresponding author; Imran Ahmad: wonder_env@yahoo.com).
